# Age-Specific Cardiovascular Risk Factors for Major Adverse Cardiac Events in Patients Undergoing Myocardial Perfusion Imaging

**DOI:** 10.3390/jcdd10090395

**Published:** 2023-09-13

**Authors:** Rosario Megna, Mario Petretta, Carmela Nappi, Roberta Assante, Emilia Zampella, Valeria Gaudieri, Teresa Mannarino, Adriana D’Antonio, Roberta Green, Valeria Cantoni, Mariarosaria Panico, Wanda Acampa, Alberto Cuocolo

**Affiliations:** 1 Institute of Biostructure and Bioimaging, National Council of Research, via T. De Amicis 95, 80145 Naples, Italy; rori.panico@ibb.cnr.it; 2 IRCCS Synlab SDN, via Gianturco 113, 80143 Naples, Italy; petretta@unina.it; 3 Department of Advanced Biomedical Sciences, University Federico II, via Pansini 5, 80131 Naples, Italy; c.nappi@unina.it (C.N.); roberta.assante@unina.it (R.A.); emilia.zampella@unina.it (E.Z.); valeria.gaudieri@gmail.com (V.G.); teresa.mannarino@unina.it (T.M.); adriana.dantonio@unina.it (A.D.); greenroby83@gmail.com (R.G.); cantoni.valeria@gmail.com (V.C.); acampa@unina.it (W.A.); cuocolo@unina.it (A.C.)

**Keywords:** cardiovascular risk factors, coronary artery disease, SPECT, myocardial perfusion imaging, MACE

## Abstract

Background: The prevalence of traditional cardiovascular risk factors shows different age-specific patterns. It is not known whether the prognostic impact of risk factors is similarly age-specific. We evaluated the profiles of cardiovascular risk factors and their prognostic impact on coronary artery disease (CAD) in relation to age. Methods: We included 3667 patients with suspected or known CAD undergoing stress myocardial perfusion imaging (MPI). We evaluated the risk for major adverse cardiac events (MACE) within three years from the index MPI in patients belonging to three groups according to age tertile distribution: <59, 59–68, and >68 years. Gender, body mass index, diabetes, hypertension, dyslipidemia, family history of CAD, smoking, angina, dyspnea, previous CAD, and MPI outcome were assessed as risk factors by a multivariable Cox’s regression. Results: The three-year risk of MACE increased progressively with age and was 9%, 13%, and 18% for each group, respectively (*p* < 0.0001). Dyspnea and abnormal MPI outcome were significant risk factors for all age groups. Diabetes and smoking were significant from the age of 59 onwards, while hypertension resulted significant for patients older than 68 years. Conclusions: The number of risk factors was significantly associated with the occurrence of MACE increase with age. It is noteworthy that a personal history of CAD was not useful for risk stratification, while MPI results were.

## 1. Introduction

The analysis of cardiovascular risk factors has multiple applications. For several decades, they are studied to update guidelines related to the prevention and care of cardiovascular diseases [1,2]. They are also included in diagnostic and prognostic algorithms to build pretest tools able to compute the probability of obtaining an abnormal cardiovascular test result as well the risk of subsequent events [3,4,5]. Demographic characteristics, such as gender and age, are risk factors usually considered in the cardiac field, together with diabetes mellitus, high blood cholesterol, hypertension, overweight, and tobacco use. On the other hand, the prevalence of risk factors can vary among different study populations and, within each of these, prevalence can again vary depending on some factors such as patients’ age [6,7,8]. The evaluation of risk obtained considering these characteristics is called risk stratification [9]. It is a technique for systematically categorizing patients based on their health status and other factors. In this way, it provided a more appropriate level of care for distinct subgroups of patients. On this matter, the Guideline on Cardiovascular Disease Prevention in Clinical Practice, recently published by the European Society of Cardiology [2], proposed a different risk stratification and recommendation for treatment according to age groups. The purpose is based on the concept of avoiding under-treatment in young patients and over-treatment in older persons [2]. Concerning this topic, in this article we propose a study on age-specific cardiovascular risk factors for major adverse cardiac events (MACE) in patients undergoing myocardial perfusion imaging (MPI). In particular, epidemiologic data show that improved control of cardiovascular risk factors has resulted in a temporal decrement in the incidence and severity of coronary artery disease (CAD) and its related mortality in the last decades [10,11,12]. However, despite a progressive decline observed [13,14,15,16,17], CAD remains one of the leading causes of mortality in developed countries [18]. In our investigation, we considered MACE within three years from the MPI exam, obtained by single-photon emission computed tomography (SPECT). This technique is commonly used for the noninvasive assessment of suspected or known CAD, and it has clinical advantages including high sensitivity, specificity, and negative predictive value for both diagnostic and prognostic purposes [19,20,21,22,23]. The prevalence of traditional cardiovascular risk factors shows different age-specific patterns [6,7,8]. However, it is not known whether the prognostic impact of risk factors is similarly age-specific. Therefore, the aim of this study was to evaluate the profiles of cardiovascular risk factors and their prognostic impact on CAD in relation to age.

## 2. Materials and Methods

### 2.1. Study Population

We considered 3667 consecutive patients of age 18 years or older undergoing 99mTc-sestamibi SPECT myocardial perfusion imaging for suspected or known CAD at our academic center between January 2004 and December 2019, available for a three-year follow-up for MACE. Based on the clinical diagnostic protocol, only patients that have anomalies in cardiac exams (ECG, echo, etc.) or have a prior history of CAD or with specific and recurrent symptoms (e.g., angina or dyspnea) can be referred to SPECT study. A patient with known CAD indicates a subject with a previous myocardial infarction and/or revascularization. A subject can also be definite with known CAD when there is evidence in a Coro-CT or invasive coronary angiography (ICA). Based on clinical evaluations, enrolled patients in our academic center were categorized as follows: (1) diagnostic evaluation; (2) post-revascularization evaluation (known CAD patients); (3) pre-surgical evaluation (patients not undergoing revascularization); (4) residual ischemia (patients at high-risk CAD).

At the time of testing, clinical teams collected pertinent demographic and clinical information, past cardiac history, and CAD risk factors based on patient reports or available medical records. MACE was defined as a composite of cardiac death, nonfatal myocardial infarction, or late coronary revascularization (including both percutaneous coronary intervention and coronary artery bypass grafting greater than 90 days following MPI), and severe heart failure requiring hospitalization. These patients were part of an ongoing prospective dedicated database [24].

### 2.2. SPECT Imaging

Patients underwent stress-optional rest 99mTc-sestamibi SPECT myocardial perfusion imaging by physical exercise or pharmacologic stress using dipyridamole, according to the recommendations of the European Association of Nuclear Medicine [25]. In all patients, beta-blocking medications and calcium antagonists were withheld for 48 h and long-acting nitrates for 12 h before testing. For patients undergoing exercise tests, symptom-limited treadmill standardized protocols were performed. For the dipyridamole stress test, patients were instructed not to consume products containing caffeine for 24 h before the test. Dipyridamole was infused at a dose of 0.142 mg × kg^−1^ × minute^−1^ intravenous over 4 min. A dose of 100 mg of aminophylline was administered intravenously in the event of chest pain or other symptoms, or after significant ST depression. At peak exercise, or 4 min after completion of dipyridamole infusion, patients were intravenously injected with 99mTc-sestamibi (8 to 10 mCi for stress and 32 to 40 mCi for rest). Imaging was started 30 to 45 min after tracer injection using a dual-head rotating gamma camera (E.CAM, Siemens Medical Systems, Hoffman Estates, IL, USA) equipped with a low-energy, high-resolution collimator and connected with a dedicated computer system. No attenuation or scatter correction was used. An automated software program (e-soft, 2.5, QGS/QPS, Cedars-Sinai Medical Center, Los Angeles, CA, USA) was used to calculate left ventricular volumes and ejection fraction and the scores incorporating both the extent and severity of perfusion defects using standardized segmentation of myocardial regions [26]. Perfusion defects were quantified by summing the scores for each segment and expressed as summed stress score, representing the total myocardium abnormal. Each myocardial segment was scored from normal (score = 0) to absent perfusion (score = 4). The summed stress score, representing the total myocardium abnormal (i.e., necrotic and ischemic tissue), was obtained by adding the scores of the segments of the stress images. A summed stress score >3 was considered abnormal.

### 2.3. Clinical Definitions

Chest pain was defined according to the American College of Cardiology/American Heart Association 2002 guideline update on exercise testing [27]. Patients were considered as having diabetes if they were receiving treatment with oral hypoglycemic drugs or insulin. A family history of CAD was defined as a diagnosis of CAD in a first-degree relative prior to or at 55 years of age. Hypertension was defined as a blood pressure > 140/90 mmHg or the use of anti-hypertensive medication. Hyperlipidemia was defined as a total cholesterol level > 6.2 mmol/L or treatment with cholesterol-lowering medication. Smoking history was defined as prior or current tobacco use. Body mass index was defined as the patient’s weight in kilograms divided by the square of height in meters. Known CAD events were defined as prior myocardial infarction, acute coronary syndrome, or revascularization.

### 2.4. Statistical Analysis

In order to obtain age-specific cardiovascular risk factors, we split our study population in tertiles of the distribution related to patient’s age. In this way, we obtained three age groups, each containing a very similar number of patients. The three groups of patients were <59, 59–68, and >68 years old. On the other hand, if we had chosen different intervals such as <60, 60–70, and >70 we would have had sizes with percentage variations of up to 60% between groups. On this topic, we would like to clarify that different sizes among groups involve a different statistical relevance in performing tests. Continuous variables were expressed as mean ± standard deviation and categorical data as percentages. Differences between age groups were analyzed by Kruskal–Wallis test or χ^2^ test, as appropriate. Trends were analyzed by a linear model and χ^2^ for trend test, respectively, for continuous and categorical variables. Kaplan–Meier survival analysis was performed to evaluate MACE with respect to age groups. For comparing the three survival curves obtained by this analysis, we computed a log-rank test. The annualized event rate was computed by the following formula:

r=−ln(1−p)/ΔT

where the probability p was obtained as the ratio between the number of patients with MACE and the total number of patients in each age group, while ΔT represent the interval of time considered. This relationship descends from the following considerations: (1) a probability represents the likelihood of an event happening over a specific period of time; (2) an instantaneous rate can be converted to a probability over a particular time period, if the rate is assumed to be constant over that period [28]. Errors on annualized event rate were computed by propagation of statistical errors for a function, which is as first derivative of the function in module multiplied by the error on the rate:

δr=|dr/dp|·ϵ(p)

with ϵ(p) assumed to be Poissonian. In our investigation, we considered the following risk factors: gender, body mass index, diabetes, hypertension, hyperlipidemia, smoking, angina, dyspnea, CAD, and MPI outcome. For each age group, these factors were used as independent variables in a Cox’s regression multivariable analysis with MACE as dependent variable. The Cox’s model is the most commonly used multivariable approach for censored survival time data analysis in medical research. By this regression model, are described the relation between the event incidence, as expressed by the hazard function and a set of covariates. A property of this statistical technique is that each variable is adjusted by removing the effect of the other covariates. We also reported the hazard ratios (HR) computed by the Cox’s analysis, which represent the increased risk of MACE in patients with a risk factor with respect to patients without that risk factor, in a considered interval of time. As post-hoc analyses, we evaluated the incremental value of MPI covariate by the likelihood ratio test (LRT), and performed the Cox’s regressions separately for patients with suspected and known CAD. In this way, we verified the contribution to the significant covariates due to the two sub-groups, which have a different risk at baseline. Two-sided *p*-values < 0.05 were considered statistically significant. Confidence intervals (CI) were computed at 95%. Overlayers at 95% CI indicate no difference among age groups for significant risk factors. Statistical analysis was performed using the R software, version 4.3.0 (The R Foundation for Statistical Software, Vienna, Austria). Supplementary R packages for pre-processing, data analysis, and graphs were *dplyr*, *survival*, *survplot*, *ggplot2*, and *ggfortify*.

## 3. Results

Table 1 summarizes the clinical characteristics of patients according to the three age groups. Our study population was in prevalence constituted by pre-surgery category, while about 5% were subjects referred to MPI exam for a diagnosis. Of note is that post-revascularization and residual ischemia categories together are approximately the known CAD patients. Therefore, in the following, we do not consider the clinical evaluation as an independent variable, whose categories are content in the CAD variable. Except for gender, we observed a significant difference for all the other variables. The prevalence trend of risk factors significantly increased with age for diabetes, hypertension, dyspnea, known prior CAD events, abnormal MPI, and stress test, while decreased for family history of CAD, smoking, and angina. Gender, body mass index, and hyperlipidemia showed no trend. The percentage of patients undergoing exercise as compared to pharmacological stress test declined with age. Conversely, the percentage of patients with an abnormal MPI results increased with age.

Table 2 reports MACE observed during the follow-up for each age group. The absolute number of patients with MACE increases significantly with increasing age, with percentages of 9%, 13%, and 18% for each of the three groups, respectively. Cardiac death determined this trend, while non-fatal myocardial infarction and revascularization decreased for older patients. No difference was detectable among the age groups for the occurrence of severe heart failure.

**Figure 1:**
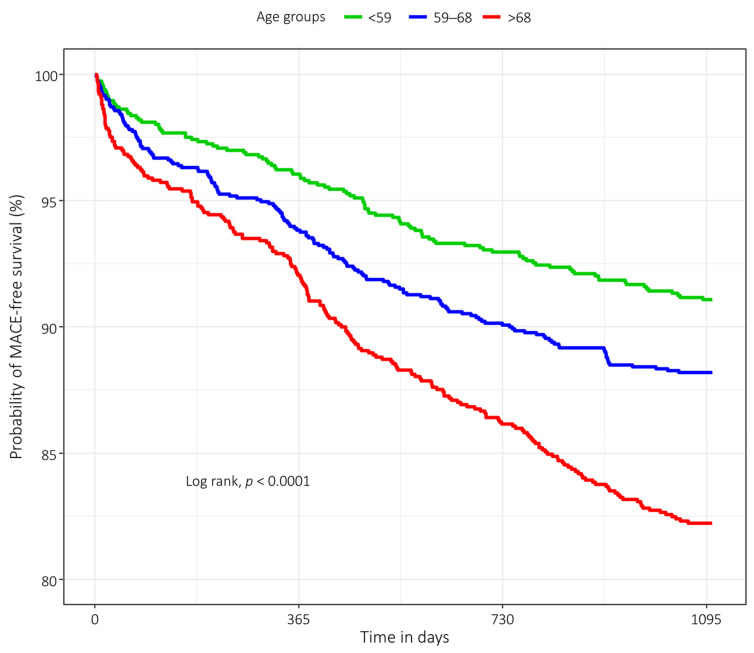
Kaplan–Meier event-free survival curves with 95% CI for the three groups of patients during the three-year follow-up period.

Figure 1 shows Kaplan–Meier curves and the 95% CI for the three groups of patients. The curves tend to separate over time, indicating a higher rate of MACE with increasing patient’s age. The log-rank *p*-value was <0.0001, confirming a different event rate on time among groups.

The annualized event rate was computed as the average for each year of observation. The event rate resulted in more than doubled from younger to older patients, increasing on average from 3.1% to 6.5% events per year (Table 3). It is noteworthy that the rate of events computed for each year of observation resulted increasing with respect to patients’ age and decreasing in time for all the groups.

**Figure 2:**
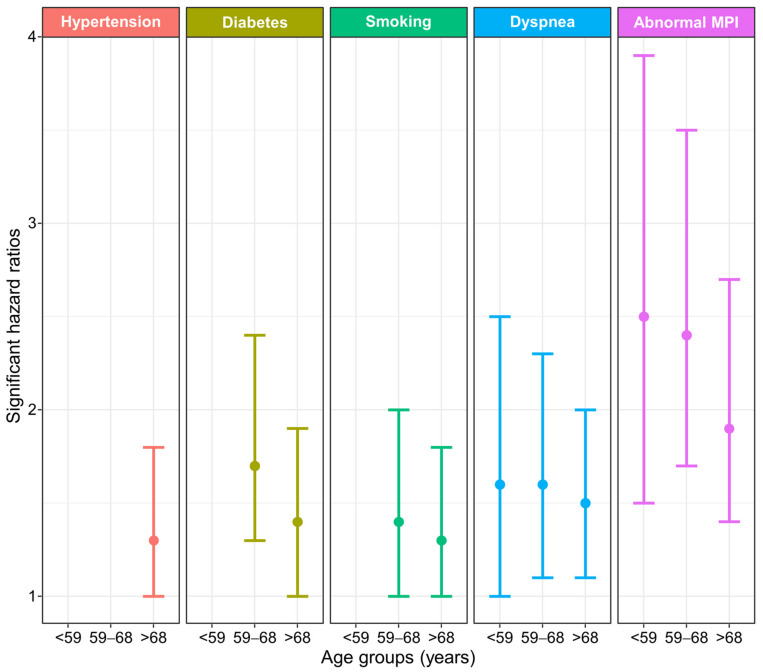
Hazard ratios with 95% CI of significant risk factors by multivariable Cox regression analysis for each age group.

Table 4 and Figure 2 summarize the Cox’s regression multivariable results computed for each age group. As shown, the number of risk factors significantly associated with the occurrence of MACE increased with patients’ age. Concerning the first age tertile, dyspnea, and abnormal stress MPI were significant. For patients belonging to the second tertile, diabetes, smoking, dyspnea, and abnormal MPI resulted significantly. The same variables were significant for patients belonging to the third tertile, with the addition of hypertension.

As a post hoc analysis, we computed the incremental value of the MPI covariate by the likelihood ratio test (LRT) related to Cox’s regressions without and with it (see Table 5).

We also split the data related to patients into two sub-groups, with suspected and known CAD, and analyzed it by Cox’s regressions according to the age groups (see Table 6).

## 4. Discussion

We evaluated the profiles of cardiovascular risk factors and their prognostic impact on the occurrence of MACE within three-year from the index examination in relation to age in 3667 consecutive patients with suspected or known CAD undergoing stress MPI. To the best of our knowledge, there are no other studies on age-specific risk factors associated with MACE in patients undergoing MPI by SPECT. Therefore, our study is the first explorative investigation on the topic. The results show an age-specific prevalence of traditional risk factors for CAD and indicate that risk factors are associated with a poor prognosis regardless of stress MPI findings and known prior CAD. Furthermore, the number of risk factors significantly associated with MACE on multivariable analysis increases with age.

Except for gender, clinical characteristics related to our study population were statistically different with respect to patients’ age. Indeed, comorbidities such as diabetes and hypertension, and symptoms such as angina, had a greater prevalence with increasing age of patients. We observed an analog increasing trend with age for the number of patients with known prior CAD and of patients with abnormal MPI results. The increasing percentage of pharmacological stress tests instead of exercise stress tests indicates a worse health condition in older patients who were not able to perform the exercise.

In our study population, the risk of MACE within three years of the MPI exam doubled with the age groups. This increase was due to cardiac deaths caused by fatal myocardial infarction— non-fatal myocardial infarction and revascularization —both decreasing for older patients. Instead, we did not observe differences for severe heart failure, indicating independence of this event type with patients’ age. Kaplan–Meier curves showed different rates of MACE on time among the three groups. In particular, those relating to the first two age intervals are at the limit of 95% CI, while that relating to the group of older patients is clearly detached from the previous one highlighting a minor survival-free MACE.

The annualized event rate reflects the probability of MACE per group, with average percentages of events close to one-third of the total events over the three-year period. It is noteworthy that the maximum rate of events for all the groups was during the first year after the MPI exam. In the following two years of observation, event rates decreased for all patients. Our finding is in agreement with the observation that the incidence of recurrent MACE is high in patients suffering from their first event, particularly during the first 6 months after the index event [29]. Therefore, the first year after referral to MPI examination, mostly due to cardiac symptoms, can be interpreted as a critical period for patients. Passed that period at higher risk, the probability of MACE decreases.

Despite the differences observed for most of the clinical characteristics, few covariates were significantly at the Cox’s regression in relationship to MACE. The MPI outcome and dyspnea were recurrent risk factors for the three age groups, while known prior CAD was never significant. Therefore, in our study population, the presence of an abnormal stress MPI result was more important than being a patient with known CAD. Indeed, the HR for patients with an abnormal MPI was approximatively double with respect to patients with a normal study. Conversely, the analysis of pooled data from six studies including patients aged ≥45 years at ≥6 months after a coronary event demonstrated that adherence to lifestyle advice and guideline-directed medical therapy could add several healthy years of life after a heart attack [30].

Strictly speaking, dyspnea cannot be considered among the traditional risk factors. However, our results confirm that it is useful for clinical decision-making for all age groups. Indeed, the HR for MACE was about fifty percent higher for patients with this symptom. On the other hand, diabetes, smoking, and hypertension resulted significantly only for older patients, with an increased risk of at least thirty percent. Interestingly, if a risk factor is significant across multiple age groups, this does not imply that the associated HRs are significantly different from each other. In other words, HR remains independent of age groups.

Post hoc analysis of the incremental value of the MPI variable showed a relevant increase of LRT for the model that contained it. In fact, this imaging variable played a fundamental role in the MACE prediction, as highlighted by Cox’s regressions according to the CAD variable. In this analysis, MPI was significant for suspected CAD. Instead, diabetes and smoking were significant for known CAD, while dyspnea was a common characteristic between the two sub-groups. Logically, the others significant HR observed in the whole study population (such as hypertension) are due to a major number of data used in the regressions. Furthermore, these evidence are useful in the characterization of patients.

The Asia Pacific Cohort Studies Collaboration in 2006 published a meta-analysis of 41 cohort studies with a total of 582,134 persons from Asia, Australia, and New Zealand. High systolic blood pressure was the most important modifiable risk factor, contributing to the excess cardiovascular risk that occurs with aging [7]. More recently, Wang et al. in 2021 published a study obtained by a multicenter, population-based, nationwide prospective cohort study with 119,455 participants included in the analysis for cardiovascular disease events. The baseline survey was conducted between 2011 and 2012, while the follow-up survey was conducted between 2014 and 2016. Their age groups were the following: 40, <55; 55, <65; 65, <75; ≥75 years old. Among risk factors in common with our study, there was significant HR diabetes for all groups, hypertension in the first three groups, and dyslipidemia and obesity only in the second group [6]. Another study on age-specific vascular risk factor profiles according to stroke subtype highlighted that the prevalence of common cardiovascular risk factors shows different age-specific patterns among various stroke subtypes [31]. The Authors suggest that these patterns may target stroke prevention efforts in specific risk groups [31].

A potential limitation of this study concerns that it was obtained from the data that constitute the experience of a single medical center. In addition, although we included patients who were part of the ongoing prospective dedicated database, this is a retrospective study with all of the limitations inherent to retrospective investigations. Thus, the results may not be applicable in other study populations, as verified in some external validations performed for cardiovascular risk models [32,33]. Data related to follow-up such as cardiac events or mortality were not available on all patients undergoing MPI, who were not included in the study. In fact, our study covers a time interval of 15 years, during which patients underwent increasingly guideline-directed effective treatments. Our study did not include variables focusing on systemic inflammation, hyperlipidemia, platelets, and coagulation pathways, which may have a role in residual cardiovascular risk, beyond traditional risk cardiovascular risk factors [34]. Finally, a possible limitation is represented by the sample size for Cox’s model. Conflicting opinions concern the classical rule published by Peduzzi et al. [35] that considers the need for 10 events per variable. However, according to Vittinghoff and McCulloch’s simulations [36], our relative bias should be a few percent, and CI coverage very close to 95%.

Studies on cardiovascular risk stratification are also performed by artificial intelligence, in particular using machine learning algorithms. Several approaches such as support vector machines, naïve Bayesian, neural networks, boosting, and other procedures are used for clinical evaluations of cardiovascular patients [37,38,39].

## 5. Conclusions

The number of risk factors was significantly associated with the occurrence of MACE increase with age. It is noteworthy that the history of CAD was not useful for risk stratification, while MPI results were. This finding might be encouraging for patients diagnosed with CAD, giving them good life expectancies if appropriately managed.

## Figures and Tables

**Table 1:** Characteristics of cohort according to age groups.

	<59 Years (*n* = 1166)	59–68 Years (*n* = 1330)	>68 Years (*n* = 1171)	*p*-Value	Trend (*p*)
Clinical evaluations					
Diagnosis, *n* (%)	40 (3)	62 (5)	64 (5)	0.06	↑ (0.02)
Post-revascularization, *n* (%)	271 (23)	327 (25)	360 (31)	<0.001	↑ (<0.001)
Pre-surgery, *n* (%)	759 (65)	795 (60)	623 (53)	<0.001	↓ (<0.001)
Residual ischemia, *n* (%)	96 (8)	146 (11)	124 (11)	0.05	↔ (0.06)
Male gender, *n* (%)	744 (64)	880 (66)	770 (66)	0.43	↔ (0.32)
Body mass index (kg/m^2^)	28.1 ± 4.4	27.8 ± 4.0	27.6 ± 3.8	0.01	↔ (0.07)
Diabetes, *n* (%)	278 (24)	510 (38)	442 (38)	<0.001	↑ (<0.001)
Hypertension, *n* (%)	774 (66)	1053 (79)	958 (82)	0.001	↑ (<0.001)
Hyperlipidemia, *n* (%)	641 (55)	827 (62)	680 (58)	<0.001	↔ (0.13)
CAD family history, *n* (%)	516 (44)	550 (41)	408 (35)	<0.001	↓ (<0.001)
Smoking, *n* (%)	450 (39)	443 (33)	288 (25)	<0.001	↓ (<0.001)
Angina, *n* (%)	419 (36)	391 (29)	357 (30)	0.001	↓ (0.005)
Dyspnea, *n* (%)	213 (18)	275 (21)	301 (26)	<0.001	↑ (<0.001)
Known prior CAD, *n* (%)	373 (32)	490 (37)	491 (42)	<0.001	↑ (<0.001)
Abnormal MPI, *n* (%)	356 (31)	473 (36)	471 (40)	<0.001	↑ (<0.001)
Exercise stress test, *n* (%)	797 (68)	779 (59)	479 (41)	<0.001	↓ (<0.001)

Increasing ↑, constant ↔, and decreasing ↓ trend.

**Table 2:** MACE according to age groups.

	<59 Years (*n* = 1166)	59–68 Years (*n* = 1330)	>68 Years (*n* = 1171)	*p*-Value	Trend (*p*)
MACE, *n* (%)	104 (9)	157 (12)	208 (18)	<0.001	↑ (<0.001)
Cardiac death, *n* (%)	19 (18)	35 (22)	89 (43)	<0.001	↑ (<0.001)
Myocardial infarction, *n* (%)	31 (30)	35 (22)	28 (14)	0.002	↓ (<0.001)
Revascularization *, *n* (%)	40 (39)	64 (41)	59 (28)	0.03	↓ (0.03)
Severe heart failure, *n* (%)	14 (13)	23 (15)	32 (15)	0.90	↔ (0.65)

Increasing ↑, constant ↔, and decreasing ↓ trend.* Interventions not scheduled by MPI study.

**Table 3:** Annual event rate according to age groups.

	<59 Years (*n* = 1166)	59–68 Years (*n* = 1330)	>68 Years (*n* = 1171)
Annual event rate, %	3.1 (0.3)	4.2 (0.4)	6.5 (0.5)
First year, %	4.0 (0.6)	6.4 (0.7)	8.3 (0.9)
Second year, %	3.1 (0.5)	3.8 (0.6)	6.1 (0.8)
Third year, %	1.9 (0.4)	1.9 (0.4)	4.0 (0.6)

In parenthesis are reported errors on rates.

**Table 4:** Cox’s regression multivariable analysis for MACE according to age groups.

	<59 Years (*n* = 1166)			59–68 Years (*n* = 1330)			>68 Years (*n* = 1171)		
	Coefficient (SE)	HR (95% CI)	*p*	Coefficient (SE)	HR (95% CI)	*p*	Coefficient (SE)	HR (95% CI)	*p*
Male gender	0.413 (0.262)	1.5 (0.9–2.5)	0.11	0.366 (0.206)	1.4 (0.9–2.1)	0.07	0.167 (0.171)	1.1 (0.8–1.6)	0.32
Body mass index	−0.012 (0.024)	0.9 (0.9–1.0)	0.61	0.006 (0.020)	1.0 (0.9–1.0)	0.75	−0.001 (0.018)	0.9 (0.9–1.0)	0.94
Diabetes	0.271 (0.216)	1.3 (0.8–2.0)	0.21	0.584 (0.164)	1.7 (1.3–2.4)	<0.001	0.368 (0.142)	1.4 (1.0–1.9)	0.01
Hypertension	0.308 (0.242)	1.3 (0.8–2.1)	0.20	0.192 (0.224)	1.2 (0.7–1.8)	0.39	0.480 (0.218)	1.6 (1.0–2.4)	0.02
Hyperlipidemia	0.121 (0.221)	1.1 (0.7–1.7)	0.58	0.220 (0.180)	1.2 (0.8–1.7)	0.22	−0.241 (0.147)	0.7 (0.5–1.0)	0.10
CAD family history	0.003 (0.202)	1.0 (0.6–1.4)	0.98	0.001 (0.168)	0.9 (0.7–1.3)	0.99	0.238 (0.145)	1.2 (0.9–1.6)	0.10
Smoking	0.387 (0.201)	1.4 (0.9–2.1)	0.05	0.370 (0.166)	1.4 (1.0–2.0)	0.02	0.317 (0.155)	1.3 (1.0–1.8)	0.04
Angina	0.223 (0.214)	1.2 (0.8–1.9)	0.29	0.072 (0.183)	1.0 (0.7–1.5)	0.69	0.073 (0.151)	1.0 (0.8–1.4)	0.62
Dyspnea	0.497 (0.231)	1.6 (1.0–2.5)	0.03	0.502 (0.187)	1.6 (1.1–2.3)	0.007	0.419 (0.152)	1.5 (1.1–2.0)	0.006
CAD	0.382 (0.236)	1.4 (0.9–2.3)	0.10	−0.187 (0.186)	0.8 (0.5–1.1)	0.29	−0.039 (0.157)	0.9 (0.7–1.3)	0.80
Abnormal MPI	0.918 (0.231)	2.5 (1.5–3.9)	<0.001	0.909 (0.183)	2.4 (1.7–3.5)	<0.001	0.690 (0.157)	1.9 (1.4–2.7)	<0.001

**Table 5:** Likelihood ratio test obtained by Cox’s regressions without and with MPI covariate, according to the age groups.

	<59 Years (*n* = 1166)	59–68 Years (*n* = 1330)	>68 Years (*n* = 1171)
**without MPI**	51.43	47.63	41.02
**with MPI**	67.61	72.36	60.44

All *p*-values associated with LRT resulted <0.0001.

**Table 6:** Cox’s regression multivariable analysis for MACE according to CAD variable and age groups.

	Suspected CAD						Known CAD					
	<59 Years (*n* = 793)		59–68 Years (*n* = 840)		>68 Years (*n* = 680)		<59 Years (*n* = 373)		59–68 Years (*n* = 490)		>68 Years (*n* = 491)	
	HR (95% CI)	*p*	HR (95% CI)	*p*	HR (95% CI)	*p*	HR (95% CI)	*p*	HR (95% CI)	*p*	HR (95% CI)	*p*
Male gender	1.3 (0.7–2.6)	0.32	1.5 (0.9–2.4)	0.07	1.3 (0.8–2.1)	0.15	1.3 (0.5–3.1)	0.53	1.1 (0.5–2.2)	0.77	0.9 (0.5–1.5)	0.84
Body mass index	0.9 (0.8–1.0)	0.09	1.0 (0.9–1.0)	0.58	0.9 (0.9–1.0)	0.41	1.0 (0.9–1.1)	0.18	0.9 (0.9–1.0)	0.85	1.0 (0.9–1.0)	0.24
Diabetes	1.3 (0.6–2.8)	0.35	1.4 (0.9–2.2)	0.08	1.4 (0.9–2.1)	0.09	1.2 (0.7–2.1)	0.39	2.4 (1.4–4.0)	***	1.4 (1.0–2.2)	*
Hypertension	0.9 (0.5–1.8)	0.99	1.2 (0.6–2.1)	0.48	1.5 (0.9–2.8)	0.10	1.8 (0.8–3.8)	0.09	1.1 (0.5–2.3)	0.72	1.7 (0.9–3.3)	0.08
Hyperlipidemia	1.0 (0.5–1.9)	0.82	1.3 (0.8–2.0)	0.23	0.8 (0.5–1.2)	0.46	0.9 (0.5–1.8)	0.98	1.1 (0.6–2.0)	0.63	0.7 (0.4–1.1)	0.15
CAD family history	0.9 (0.5–1.7)	0.83	0.9 (0.6–1.5)	0.94	1.3 (0.8–1.9)	0.17	1.0 (0.5–1.7)	0.94	1.0 (0.6–1.7)	0.86	1.2 (0.8–1.8)	0.34
Smoking	1.7 (0.9–3.1)	0.06	1.2 (0.7–1.8)	0.42	1.3 (0.8–2.0)	0.22	1.2 (0.7–2.1)	0.36	1.7 (1.1–2.9)	*	1.4 (0.9–2.1)	0.10
Angina	1.1 (0.6–2.2)	0.58	1.0 (0.6–1.6)	0.95	0.9 (0.6–1.4)	0.85	1.2 (0.6–2.2)	0.47	1.1 (0.6–2.0)	0.54	1.1 (0.7–1.8)	0.41
Dyspnea	1.6 (0.8–3.3)	0.14	1.0 (0.6–1.8)	0.77	1.8 (1.2–2.7)	**	1.7 (0.9–3.2)	0.07	2.6 (1.5–4.4)	***	1.2 (0.8–1.9)	0.29
Abnormal MPI	4.7 (2.5–8.6)	***	3.4 (2.2–5.4)	***	2.5 (1.7–3.8)	***	1.3 (0.7–2.3)	0.31	1.5 (0.8–2.5)	0.13	1.4 (0.9–2.2)	0.10

*** < 0.001; ** < 0.005; * < 0.05.

## Data Availability

The data presented in this study are available on request from the corresponding author. The data are not publicly available due to privacy reasons.

## Abbreviations

MACE	major adverse cardiac events
MPI	myocardial perfusion imaging
CAD	coronary artery disease
SPECT	single-photon emission computed tomography
LRT	likelihood ratio test
CI	confidence intervals
